# Dynamic Effects of Immersive Bilingualism on Cortical and Subcortical Grey Matter Volumes

**DOI:** 10.3389/fpsyg.2022.886222

**Published:** 2022-04-25

**Authors:** Lidón Marin-Marin, Victor Costumero, César Ávila, Christos Pliatsikas

**Affiliations:** ^1^Neuropsychology and Functional Neuroimaging Group, Department of Basic Psychology, Clinic and Psychobiology, Universitat Jaume I, Castelló de la Plana, Spain; ^2^School of Psychology and Clinical Language Sciences, University of Reading, Reading, United Kingdom; ^3^Centro de Investigación Nebrija en Cognición, Universidad Nebrija, Madrid, Spain

**Keywords:** bilingualism, neuroplasticity, grey matter, volume, immersion, dynamic, non-linear

## Abstract

Bilingualism has been shown to induce neuroplasticity in the brain, but conflicting evidence regarding its specific effects in grey matter continues to emerge, probably due to methodological differences between studies, as well as approaches that may miss the variability and dynamicity of bilingual experience. In our study, we devised a continuous score of bilingual experiences and we investigated their non-linear effects on regional GM volume in a sample of young healthy participants from an immersive and naturalistic bilingual environment. We focused our analyses on cortical and subcortical regions that had been previously proposed as part of the bilingual speech pipeline and language control network. Our results showed a non-linear relationship between bilingualism score and grey matter volume of the inferior frontal gyrus. We also found linear increases in volumes of putamen and cerebellum as a function of bilingualism score. These results go in line with predictions for immersive and naturalistic bilingual environments with increased intensity and diversity of language use and provide further evidence supporting the dynamicity of bilingualism’s effects on brain structure.

## Introduction

Bilingualism-the experience of being exposed to two languages and manage them in everyday life-has been shown to induce neuroplasticity in the brain (Grundy et al., 2017). During language production, bilinguals need to select one language and suppress the other, while adequately articulating the target language, which results in increased demands for linguistic control and, consequently, in changes in brain structure and function to accommodate these heightened demands (Tao et al., 2021). Different models have attempted to describe the location and characteristics of these changes, and the particular features of the bilingual experience that contribute to them. For instance, the Adaptive Control Hypothesis (ACH) proposed that any effects of bilingualism on brain structure are dependent on the interactional context in which the individual uses their languages and the specific control processes that different contexts entail: *single language contexts* in which languages are used separately in different environments; *dual-language contexts* in which both languages are used but separately with different speakers; and *dense code-switching contexts* where speakers use both languages interleaving them in their discourse (Green and Abutalebi, 2013). Based on previous evidence, they propose a brain network for language control and speech, composed by inferior frontal, parietal, anterior cingulate, motor and premotor cortices, thalamus, caudate nucleus, putamen, cerebellum and insula (Abutalebi and Green, 2016). These regions are hypothesized to be differentially affected by bilingual experience depending on the interactional context: while dense code-switching contexts would engage more the cerebellum and left inferior frontal cortex, dual or multiple language interactional contexts would engage bilateral inferior frontal, anterior cingulate and parietal cortices, caudate nucleus, putamen, and the thalamus. Other authors have proposed that a brain adaptation pattern arises with increased *length* of immersion in bilingual environments, characterized by an initial tissue volume increase in frontostriatal regions, followed by reductions in volume and lower functional recruitment of frontal executive regions, as well as greater recruitment and further expansions of posterior and subcortical areas, a phenomenon they call the “bilingual anterior-to-posterior and subcortical shift” (BAPSS; Grundy et al., 2017). However, mixed evidence regarding the specific brain changes produced by bilingual *experience* continues to emerge. Namely, when investigating grey matter (GM) differences between bilinguals and monolinguals, the former generally show higher volume, density and cortical thickness, as well as shape expansions, in cortical, subcortical and cerebellar areas, but some studies have also found results in the opposite direction – lower volumes in bilinguals—or no differences at all between the groups [see Tao et al. (2021) for a systematic review].

Apparent inconsistencies between studies when investigating bilingualism and GM structure may stem from multiple sources. Methodological issues—e.g., the use of different measures—and sample differences have been suggested as the main origins of variation (García-Pentón et al., 2015). In fact, many investigations carried out to date used samples of bilinguals with very distinct characteristics. While some studies only considered simultaneous bilinguals—that is, bilinguals who first learned both languages at the same time (Burgaleta et al., 2016), others only included bilinguals who were not simultaneously exposed to both languages but acquired the second language (L2) early in life (Olulade et al., 2016), or late sequential bilinguals whose age of acquisition (AoA) of L2 was greater than seven (Pliatsikas et al., 2017; Deluca et al., 2019a). Moreover, the age cutoffs for different groups of bilinguals—simultaneous, early or late—are arbitrary and sometimes differ between studies (Mechelli et al., 2004; Ressel et al., 2012; Klein et al., 2014), which adds to the confusion. Levels of immersion in L2 also remarkably vary between studies, with some investigations comparing monolinguals to proficient bilinguals that frequently use L2 (Deluca et al., 2019a), while others investigate non-immersed bilinguals (Korenar et al., 2021). The Unifying the Bilingual Experience Trajectories (UBET) framework (DeLuca et al., 2020), which brings together previous models on the trajectory of neurocognitive adaptations due to bilingualism, emphasizes that different characteristics of bilingual experiences—intensity and diversity, language switching, relative proficiency, and duration—lead to adaptations in efficiency and control demands that have different consequences on cognition and brain structure. In particular, they hypothesize that increased duration and a balanced proficiency between the languages will increase *efficiency*, associated with increases in GM volume of subcortical and posterior regions, and return to baseline volumes in cortical areas that had expanded in previous initial stages of the bilingual experience. They also propose that increases in diversity, intensity, and language switching will increase *control demands*, resulting in GM volume increases in areas involved in control processes such as the inferior frontal gyrus (IFG), anterior cingulate cortex (ACC) or inferior parietal lobule, as an adaptation to these demands. Moreover, they draw attention to the consequences of the socio-linguistic environment on the interaction between bilingualism characteristics and their consequences. For instance, in countries where only one language is official and widely used in society, the use of a second one will probably be restricted to specific community contexts, which might result in compartmentalized usage of languages, different language proficiency levels, or low levels of immersion (Vaughn et al., 2019; Claussenius-Kalman et al., 2020). This type of bilingual use would be expected to require increased executive control demands whenever the least practiced language is used, with more recruitment of frontal and cortical structures, in contrast to environments with a balanced use of languages and opportunities of intense immersion, which are expected to shorten the latency by which efficiency effects materialize (DeLuca et al., 2020).

Crucially, many studies that investigated effects of bilingualism on GM structure treated bilingualism as a categorical variable, an approach that has been recently challenged (Luk and Bialystok, 2013; Anderson et al., 2018; Deluca et al., 2019a; Pliatsikas et al., 2019). When dividing participants in two groups based on their experience with languages and treating each group as a homogeneous category, relevant bilingual variability within the groups is likely missed (Grundy et al., 2017), since few people have “pure” and indistinguishable monolingual and bilingual experiences (Luk and Bialystok, 2013). Consequently, it has been argued that bilingualism would be better described as a continuum arising from bilingual experience-based factors, since these show *when* bilingualism starts to influence the system and *how* it interacts with it (Deluca et al., 2019a). Following up on the criticism on the categorical approach, recent studies have started to investigate the effects of *quantified* bilingualism on GM structure, reporting effects such as significant correlations between length of L2 immersion and globus pallidus expansions (Pliatsikas et al., 2017), and reshaping of left thalamus and right caudate nucleus volumes and decreases in left middle temporal gyrus as a function of amount of exposure to L2 (Burgaleta et al., 2016). To investigate similar effects, recent studies have looked at how structural changes can be predicted by bilingualism composite “scores” provided by tools such as the Language and Social Background Questionnaire [LSBQ, Anderson et al. (2018)], the Language Experience and Proficiency Questionnaire [LEAP-Q, Kaushanskaya et al. (2020)], and the Language History Questionnaire [LHQ3, Li et al. (2020)], all of which measure bilingualism experience-based factors such as language proficiency, AoA, or frequency of use in different contexts. For example, Deluca and colleagues (Deluca et al., 2019a) used as predictors of brain change scores derived from the LSBQ, including L2 use in social/community settings, and in home settings, as well as L2 AoA and length of immersion. Results showed that L2 AoA positively correlated with GM expansions in the left nucleus accumbens and bilateral thalamus, length of L2 immersion predicted reshaping in right caudate nucleus, expansions in right putamen and contractions in bilateral thalamus and nucleus accumbens, and social use also predicted significant expansions in left caudate nucleus, left nucleus accumbens and right thalamus. Other investigations have also found significant relationships between specific aspects of the bilingual experience and GM structure, such as negative correlations between both AoA and current exposure to L2 and GM volume in right IFG (Wei et al., 2015), reductions in left thalamus and right caudate nucleus, but expansions in left middle temporal gyrus, as a function of amount of time listening and speaking the dominant language (Burgaleta et al., 2016), and positive correlations between expansions in right globus pallidus and length of immersion in a country where L2 is dominant (Pliatsikas et al., 2017). Interestingly, another study found accent scores to be significantly correlated with GM volume in left putamen only in sequential bilinguals–the more native-like they sounded, the more left putaminal volume they showed (Berken et al., 2016). Similar patterns have been reported in studies looking at the relationship between WM integrity and AoA of L2 (Nichols and Joanisse, 2016; Rossi et al., 2017), length of L2 training/immersion (Mamiya et al., 2016), and L2 proficiency (Nichols and Joanisse, 2016; Singh et al., 2018). Taken together, this evidence suggests that the relationship between bilingual experience and brain changes may be better grasped by approaches that quantify the bilingual experience rather than more traditional categorical descriptions of bilingualism.

However, it still remains the case that even investigations that used correlational approaches might fail to describe the full patterns underlying bilingualism-induced neuroplasticity because of the use of linear approaches. These approaches assume continuous growth or reduction of brain structures as a function of bilingual experience, which is an unlikely pattern due to the mixed findings of multiple bilingualism studies (Tao et al., 2021); indeed, theories on experience-based neuroplasticity have assumed non-linear volumetric changes in the brain, with volumetric increases during skill acquisition followed by decreases that suggest efficient brain reorganisation (Lövdén et al., 2013). Therefore, non-linear approaches may be better suited to describe the changing tendencies of brain adaptations along the bilingual experience. The Dynamic Restructuring Model (DRM), a recent proposal that attempts to coherently merge all the apparently inconsistent evidence, describes bilingualism’s effects on brain structure as dynamic and non-linear, that is, following patterns of expansion and renormalization (Pliatsikas, 2020). Specifically, the DRM proposes three main stages of bilingual experience, characterized by different brain adaptations: initial exposure, consolidation, and peak efficiency. At the initial exposure stage, the model proposes that cortical GM volumes increase especially in anterior regions related to executive control, and parietal and temporal areas related to specific aspects of language learning. Subcortical and cerebellar GM volumes are also proposed to expand in this stage, due to the increases in demands for language control and selection between motor programmes. These expansions revert and renormalize cortically in the consolidation stage, potentially due to the optimization of lexical learning and control through the elimination of redundant local connections and conservation of only the most efficient. Still, cerebellar and subcortical regions continue increasing in volume, since bilinguals still need to exert language control and selection. The last stage, which is described by the author as the most difficult to characterize due to the scarcity of evidence, would be distinguished by further cerebellar increases, renormalization of the caudate nucleus and stabilization of the putamen and globus pallidus.

Notably, a recent study investigating young healthy bilinguals provides evidence in support of these non-linear patterns of GM changes (Korenar et al., 2021). Korenar and colleagues used generalized additive mixed models (GAMMs) to investigate non-linear effects of bilingual experience, as measured by a composite score that is calculated by the LSBQ (Anderson et al., 2018), on regional subcortical volumes. They found linear volume increases in putamen and thalamus as a function of bilingualism, but non-linear patterns of expansion-renormalization in bilateral caudate nuclei and expansion-plateauing in the nucleus accumbens. These results were interpreted in terms of the DRM predictions (Pliatsikas, 2020): the continuous increase in volume for putamen and thalamus goes in line with the constant need for bilinguals to select motor programmes of the target language and exert cognitive control, whereas the observed pattern in caudate nucleus reflects its central role in lexical control and selection, crucial in initial stages of bilingual experience, but likely optimised as experience increases. Moreover, the pattern observed in nucleus accumbens is interpreted to reflect the initial reward in pursuing social interactions that might reach a plateau when bilinguals reach language efficiency. Nevertheless, this study focused only on subcortical structures, and investigated a very specific sample of bilinguals: highly proficient non-immersed speakers of an L2 and with limited opportunity for active naturalistic bilingual language use. Thus, it remains to be determined whether non-linear bilingualism’s effects on brain structure extend to cortical regions and to populations with more sustained long-term immersive bilingual experiences.

In the present study, our main objective was to investigate non-linear effects of bilingual experiences on the GM structure in a healthy sample of bilinguals from the region of València. Both Spanish and Catalan are official languages widely used in society in that region, so bilinguals have the opportunity to use both of them in an active and naturalistic context. Our sample presented a wide variety of bilingual experiences, ranging from simultaneous immersed to late non-immersed bilinguals, in order to fully capture the variability of bilingual experiences and their dynamic effects. We developed a bilingualism score from a questionnaire that was appropriate to the particular linguistic environment of our participants, and this score was used as a predictor of grey matter volume in specific regions. Following up on recent work (Pliatsikas et al., 2020; Korenar et al., 2021), we used GAMMs to account for non-linear volumetric effects of bilingualism, by focusing on the regions of the speech pipeline and language control network proposed in the ACH (Green and Abutalebi, 2013). This method enabled us to model complex patterns of GM volume changes as a function of bilingual experiences, which constitutes one of the main strengths and novel aspects of our investigation, as opposed to previous studies that used categorical and linear approaches. This also allows us to account for non-linear GM changes due to age, previously described to follow an inverted U shape of initial volume increases during childhood, followed by abrupt reductions in adolescence and more stable pruning during adulthood (Giedd et al., 1999). For example, such patterns have been documented in the parietal lobe, also extending to medial and superior frontal cortices, the cingulum, postcentral cortex and occipital lobe (Tamnes et al., 2010). These patterns have been reported to differ between bilinguals and monolinguals during childhood and adolescence, with bilinguals showing less age-related reductions of frontal and parietal regions (Pliatsikas et al., 2020). Following up on previous investigations (Burgaleta et al., 2016; Pliatsikas et al., 2017; Deluca et al., 2019a; Korenar et al., 2021), we expected to find linear increases in GM volume of putamen, thalamus and cerebellum as a function of bilingualism score, as well as increases followed by reductions in the caudate nucleus. Due to the characteristics of the immersive bilingual environment of our sample, where a balanced used of the two languages is common, and in line with previous models’ predictions (Grundy et al., 2017; DeLuca et al., 2020; Pliatsikas, 2020), we expected to expand on previous evidence (Korenar et al., 2021) by finding volume increases in cortical areas—IFC, ACC, and parietal cortex—as a function of bilingualism score, accommodating for the continuous control demands exerted by a context of high diversity and intensity of use, but also a shortened latency for the return to baseline volumes due to increasing efficiency.

## Materials and Methods

### Participants

Data from 334 healthy participants was included in this study (147 females; 187 males; mean age = 23, SD = 6, range = 18–53). All participants were right-handed, had normal or corrected-to-normal vision, and reported no previous history of neurological, psychiatric or language disorders. All participants were born in Spain and living in the region of València at the time of testing. This is a territory where both Catalan and Spanish are taught during formal education and co-officially used in public administration. Since both languages are understood by most of the population (Generalitat Valenciana. Direcció General de Política Lingüística i Gestió del Multilingüïsme, 2015), a person can choose to use one or the other depending on the context, motivated by factors such as personal preferences, habits or perceived command on the languages of the interlocutor and oneself. As a consequence, participants in our sample spoke fluently only Spanish or Spanish and Catalan, and lived a complex variety of bilingual experiences, close to being “monolingual” and at different degrees of “bilingual.” This means that some of them had simultaneously acquired Spanish and Catalan (46%), while others acquired the second language later in life (54%). Moreover, some of them had a balanced use of both languages to different degrees (46%), which entailed different degrees of immersion in Catalan (years of immersion range = 0–52), while others were clearly exposed to one language over the other in their daily lives (64%).

Data from 60 of our participants had already been used in a previous study (Burgaleta et al., 2016) that serves as basis for our investigation. Therefore, this data was only used for the extraction of the bilingualism score based on our bilingualism questionnaire (See Data analysis—Bilingualism score) and subsequently excluded from further analyses, resulting in a final sample of 274 subjects (115 females; 159 males; mean age = 23, SD = 6, range = 18–53; 45,7% of simultaneous bilinguals, 42,6% immersed, 67,4% non-immersed; years of immersion range = 0–49).

Written informed consent before scanning was obtained from each subject and they received monetary compensation for their time and effort. The study was approved by the Ethics Committee of the Universitat Jaume I.

### Bilingualism Questionnaire

To assess the characteristics of the bilingual experiences of our participants, they were administered an in-house questionnaire. This questionnaire contained two sections. In the first one, demographic information was gathered, and participants were asked about their proficiency (from 1 = perfect, to 4 = very low), general frequency of use in percentages and AoA of Catalan and Spanish. In the second part, information regarding frequency of use (proportion of Spanish/Catalan use) in specific contexts (home, school, and others) and periods of time (childhood, adolescence, adulthood) was gathered (see Supplementary Material for original questionnaire and a translation into English). This resulted in a comprehensive collection of information regarding lifelong bilingual experiences of the participants in our sample.

### Magnetic Resonance Imaging Data Acquisition

Images were acquired on a 1.5-T Siemens Avanto scanner (Erlangen, Germany). Participants were placed inside the scanner in the supine position, and their heads were immobilized with cushions. Whole-brain 3-D images were collected for 6 min using a T1-weighted MPRAGE sequence, with the following parameters: TE = 3.8 ms; TR = 2200 ms; flip angle = 15°; matrix = 256 × 256 × 160 mm; voxel size = 1 mm^3^.

### Data Analysis

#### Image Preprocessing

All analyses were performed using the standard preprocessing pipeline of CAT12 (Computational Anatomy Toolbox; C. Gaser, Jena University Hospital, Jena, Germany^1^). After an initial bias correction of intensity non-uniformities, individual volumes of GM, WM, and cerebrospinal fluid were estimated applying the standard segmentation procedure of the toolbox, and images were registered to the template provided. Then, to study region-specific volumetric differences, region of interest (ROI) analysis implemented in CAT12 was performed. In this analysis, also called region-based morphometry (RBM), an anatomical atlas is transformed into native subject space, and the sum of the local GM inside the ROIs of the atlas is estimated. We restricted our analysis to the language control and speech production network proposed in the ACH (Green and Abutalebi, 2013), including IFG, ACC, parietal, motor and premotor cortices, thalamus, caudate, putamen, cerebellum and insula (see Table 1 for mean volumes of ROIs by hemisphere). Volumes of all ROIs were extracted using the LONI Probabilistic Brain Atlas [LPBA40; Shattuck et al. (2008)] provided by the toolbox, except for left and right cerebellum, thalamus and ACC, extracted using the Computational Brain Anatomy (CoBrA) atlas^2^ and the automated anatomical labelling atlas 3 [AAL3; Rolls et al. (2020)], because these subdivisions were not defined in the LPBA40. Finally, total intracranial volume (TIV) was estimated.

**TABLE 1:** Mean and standard deviation of grey matter (GM) volumes (cm^3^) of our region of interests (ROIs).

	Mean GM volume (Standard deviation)	
	Left hemisphere	Right hemisphere
Inferior frontal gyrus	24.78 (2.96)	25.41 (3.05)
Parietal (supramarginal gyrus)	8.94 (1.10)	8.52 (1.09)
Anterior cingulate cortex	51.74 (11.78)	44.32 (10.34)
Precentral gyrus	12.53 (1.46)	12.40 (1.36)
Middle frontal gyrus	24.78 (2.96)	25.41 (3.05)
Thalamus	4.58 (0.47)	4.94 (0.50)
Caudate	3.68 (0.44)	3.55 (0.42)
Putamen	4.61 (0.53)	4.60 (0.52)
Cerebellum	50.04 (4.90)	51.13 (4.97)
Insula	6.55 (0.70)	6.50 (0.74)

#### Bilingualism Score

In order to obtain a single score that reflected the degree of bilingualism of our participants, an exploratory factor analysis (EFA) was carried out from the data obtained in our bilingualism questionnaire, following the procedure used in a previous study (Anderson et al., 2018).

All analyses were performed using Rstudio (R version 3.6.3). First, a matrix of correlations was estimated between the 41 bilingualism items in our questionnaire, using *mixedCor* function from the *psych* package. Eighteen items fulfilled the criterion of correlating higher than *r* = 0.3 or lower than *r* = −0.3 with more than 50% of the rest of the items of the questionnaire. This implied discarding items related to Spanish proficiency (understanding, reading, writing, listening and fluency), probably due to the low variability in these scores found in our sample (e.g., for Spanish comprehension, mean = 1.03, SD = 0.18). A first EFA was carried out using the correlation matrix of those 18 items, and the inspection of their loadings led to the exclusion of 4 more, since they could not be clearly associated to a single factor (they were found to load strongly or very similarly in more than one). After this, 14 items were left to be analyzed (see Table 2). The Kaiser-Meyer-Olin (KMO) test (Kaiser and Rice, 2016) verified the sampling adequacy for our analysis (KMO = 0.92) and all the individual KMO values for the items were higher than 0.85. Bartlett’s test for sphericity indicated that correlations between our items were sufficiently large for factor analysis [χ^2^_(91)_ = 6759.24, *p* < 0.001], and we got an alpha of 0.97, indicating a high internal consistency of the items in our questionnaire.

**TABLE 2:** Standardized loadings of each item and factor, as a result of our exploratory factor analysis (EFA), with the strongest loading for each item indicated in bold.

	Use at home	Proficiency and use at school	General use in other contexts
% Of time hearing Cat	0.16	−0.21	**0.94**
% Of time hearing Sp	–0.08	0.16	**-0.91**
Cat/Sp use at home–child	**0.83**	0.19	0.02
Cat/Sp use at school–child	0.06	**0.59**	0.24
Cat/Sp use at home–adolescent	**0.82**	0.11	0.12
Cat/Sp use at school–adolescent	–0.14	**0.65**	0.41
Cat/Sp use at home–adult	**0.82**	0.09	0.15
Cat/Sp use at workplace–adult	–0.10	0.27	**0.61**
Cat/Sp use another context–adult	0.03	0.23	**0.64**
Writing in Cat	–0.01	**1.01**	–0.07
Pronunciation in Cat	0.18	**0.87**	–0.05
Fluency in Cat	0.25	**0.84**	–0.06
Reading in Cat	0.02	**1.03**	–0.10
Understanding of Cat	0.08	**0.95**	–0.07

*% = percentage, Cat = Catalan, Sp = Spanish.*

Next, a parallel analysis was performed using the matrix of correlations of the remaining 14 items, in order to determine the number of factors to be retained in the EFA. The output and scree plot suggested three factors. An EFA was carried out using an ordinary-least-squares minimum residual approach and an oblique rotation (*promax*), obtaining three factors and its factor loadings (Table 2). The three factors in combination explained 85% of the variance. Inspection of the distribution of the loadings revealed that Factor 1 is related to use of Catalan and Spanish at school and Catalan proficiency, Factor 2 reflects general use of both languages in contexts outside home and school, and Factor 3 represents use at home.

After obtention of factor structure, scores for each of the factors were calculated using *factor.scores* function in R and using the Harman method, which finds weights based on “idealized” variables (Grice, 2001). Lastly, a composite bilingualism score was computed by summing the factor scores weighted by each factor’s variance (Anderson et al., 2018). The final score ranged from –1.25 to 0.67 (SD = 0.47, skewness = −0.987, kurtosis = 0.127; see Supplementary Material for a graphical representation of the distribution). We verified the meaning of our score by exploring its relationship with the items of our questionnaire and found that the higher bilingual scores were present in the participants who reported a more balanced use of Catalan and Spanish, as well as balanced proficiency (high proficiency in both languages), while lower scores were found in the participants who reported unbalanced use and lower Catalan proficiency. Thus, our general bilingualism score reflects lifelong balanced use of both languages and proficiency. It is also important to note that one of the factors that forms our composite score contains proficiency in Catalan, since a balanced use of both languages at school (a significant amount of school hours in Catalan, at least 30%) is relevantly related to perception of proficiency on that language, as opposed to proficiency in Spanish, which shows little variation in scores due to its dominant role in society, expressed in specific contexts such as speaking to new people, in department stores or when using social networks (Generalitat Valenciana. Direcció General de Política Lingüística i Gestió del Multilingüïsme, 2015). Finally, our score might be reminiscent of language entropy (Gullifer and Titone, 2020) in that it measures the amount of balance between languages, but it also contains information regarding balance in proficiency and lifelong use.

#### Statistical Analysis

Data were analysed using R (version 3.6.3.)^3^, applying GAMMs by using *gam()* function of the mgcv package (Wood, 2011). GAMMs are generalized linear mixed models with linear predictors that involve a sum of smooth functions of covariates or splines (Wood, 2017)—i.e., the linear component of the model is replaced with an additive component (Hastie and Tibshirani, 1995), allowing to model non-linear data. These splines are only applied if there is enough evidence for a curve in the data, since wiggliness (number of curves) penalizes the estimated model fit. GAMMs compute the estimated degrees of freedom (edf), which indicate whether the predictor is in a non-linear (edf > 1) or a linear relationship (edf = 1) with the dependent variable. We ran a series of GAMMs in order to investigate the effects of individual bilingual experiences as measured by our bilingualism composite score on GM of each one of our ROIs.

In a first-level analyses, we used GAMMs in which we fitted a regression spline for the main effect of bilingualism score on GM volume of each ROI, with participant as a random effect, and also considering the main effect of TIV in order to control for the different head sizes of our participants. We examined the interaction effect of bilingualism score and age on GM volumes, due to the large age range in our sample and accounting for non-linear brain changes related to age and bilingualism that have been previously reported (Pliatsikas et al., 2020). We also included the interaction of bilingualism score and hemisphere in our analyses, to account for previous evidence of lateralized bilingualism effects (Deluca et al., 2019a). To do so, following up on previous studies (Pliatsikas et al., 2020; Korenar et al., 2021), we included hemisphere in our models as an ordered factor with two levels (left-right) and we ran two GAMMs, each one with one hemisphere level as reference. The interaction effect between bilingualism score and hemisphere would only be considered reliable if significant in both models.

In a second-level analyses, we analyzed the main effect of bilingual score on GM volumes collapsed across hemisphere, due to the lack of significant interactions with this variable at the first level, and including age, hemisphere and TIV as covariates. We also included participant as a random effect.

For all our results, we considered *p* < 0.05 as a threshold of significance, after correcting for family-wise error rate (FWE) using the Bonferroni correction.

#### Assessment of Model Fits

In order to assess the model fits of all the second-level models, we used the gam.check() function mgcv (Wood, 2011). All the final models converged with six to nine iterations, and the number of functions which gave rise to the regression splines were in all cases higher than the estimated degrees of freedom. For all variables of interest, *p*-values above the 0.05 significance threshold there were obtained, and the k-index was in all cases close to or above 1, which suggests that there were no significant or missed patterns in the residuals of the models (Wood, 2017). See Tables in Supplementary Material for details.

## Results

In the first-level analyses, we found that neither the interaction between bilingualism score and hemisphere nor between bilingualism and age were significant predictors in any of the ROI volumes (see Supplementary Material). Consequently, we carried out our second-level analyses collapsing the data across hemisphere for all ROIs and including hemisphere and age as covariates of no interest.

**FIGURE 1:**
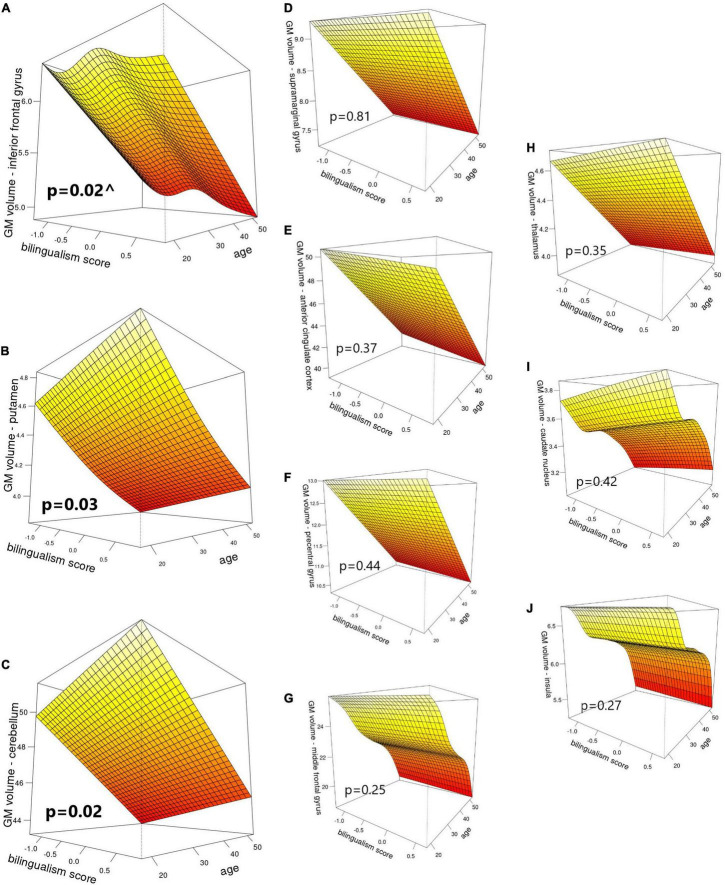
Visual representation of bilingualism score and age as predictors of GM volumes (cm^3^) in: **(A)** inferior frontal gyrus, **(B)** putamen, **(C)** cerebellum, **(D)** supramarginal gyrus, **(E)** anterior cingulate cortex, **(F)** precentral gyrus, **(G)** middle frontal gyrus, **(H)** thalamus, **(I)** caudate nucleus, and **(J)** insula. *P*-values correspond to the main effect of bilingualism score. ^ indicates edf > 1, denoting a non-linear effect.

In the second-level analyses, bilingualism score emerged as a significant predictor of GM in three structures: putamen (*p* = 0.034, FWE corrected), cerebellum (*p* = 0.018, FWE corrected) and IFG (*p* = 0.021, FWE corrected). Specifically, putaminal and cerebellar volumes showed linear increases as a function of increasing bilingual experiences. For GM volume in the IFG, bilingualism emerged as a non-linear predictor that showed an initial decrease, followed by an increase in the middle part of the bilingualism spectrum, and a final decrease at the end of the continuum, resulting in an “S” shaped distribution (see Figure 1 for details). Hemisphere emerged as a significant predictor of GM volumes of all regions except for insula, putamen and precentral gyrus, and TIV and age emerged as significant predictors for all ROIs (*p* < 0.05, FWE corrected; see Figure 1 for details).

## Discussion

In the present study, we investigated the effect of quantified bilingual experiences on regional GM volumes. To do so, we focused on a healthy sample of bilinguals living in a society where both Spanish and Catalan are actively used, in contrast to environments where languages are used in more compartmentalized manner (Vaughn et al., 2019; Claussenius-Kalman et al., 2020). Due to the language use characteristics of this environment, our sample included a wide variety of bilingual experiences, from simultaneous highly immersed to late bilinguals with little exposure to L2. In order to fully capture this variety, we considered bilingualism as a continuum, avoiding the use of two separate categories for our participants—i.e., “bilinguals” and “monolinguals.” We developed a bilingualism score from data of language use and proficiency, following up from previously published methods (Anderson et al., 2018). Finally, we used non-linear models in order to account for dynamic effects of bilingualism on GM volumes, that is, expansion and renormalization patterns (Korenar et al., 2021), in a series of regions that have been implicated in bilingual language control (Abutalebi and Green, 2016). We found a non-linear relationship between our bilingualism score and GM volume in the IFG; specifically, in the lower and higher parts of the continuum of bilingual experiences, there was a decrease of volume as a function of bilingualism, while we found increases in the middle part of the continuum. We also found that GM putaminal and cerebellar volumes increased linearly as a function of bilingualism. None of these effects interacted with hemisphere, and no other significant effects were observed. The next paragraphs will elaborate on the significant findings and discuss them in the context of similar effects that have been reported in the literature.

The IFG is one of the core cortical areas implicated in language control (Abutalebi and Green, 2016), and its GM volume has been shown to increase in L2 learners with brief experience–3 weeks to 4 months–compared to monolinguals (Stein et al., 2012; Hosoda et al., 2013; Legault et al., 2019). Based on these findings, the IFG was one of the cortical regions predicted to increase its volume in initial stages of bilingualism and later renormalize as duration of bilingual experience increases (Grundy et al., 2017; Pliatsikas, 2020). This suggestion partly matches the pattern of our current findings: The volume reductions we found in IFG at the lower end of the bilingualism continuum could be explained by the characteristics of our sample: immersed bilinguals with such limited bilingual experiences could be considered “passive bilinguals” (Calabria et al., 2020; Costumero et al., 2020), i.e., they have been exposed to a second language and are able to understand it, but currently have limited opportunities to use it and/or switch between languages. Thus, IFG might have increased its volume at an earlier point of their bilingual experience and renormalization might be already in place as the opportunities to use both languages start to increase. This would also go in line with recent evidence showing that forced switching implies increased brain activity in right IFG as measured by magnetoencephalography (MEG), an effect that is absent during natural switching (Zhu et al., 2022). Given the bilingual characteristics of the region where we conducted our study, where a big majority of the population is able to understand both languages, switching is probably more natural than enforced by the context—if the interlocutor understands both languages, changes from one to the other can be performed freely, not because they are required for successful communication. Therefore, reductions in IFG volume might be related to an increase in experience with naturalistic switching and reduced involvement of the IFG. It should be noted that we did not ask our participants if they performed forced or natural switching, so this limits our interpretation. Finally, the UBET predicts that increased intensity and diversity of language use will reduce the latency by which efficiency adaptations and automation happen as a function of duration of use (DeLuca et al., 2020). Our study was carried out in an environment where two languages are broadly used and opportunities for interacting using both are plentiful, which might increase and diversify the exposure to L2 in the earliest stages of acquisition of the language and accelerate the process of optimisation and pruning of GM cortical volumes.

Our results also showed an unexpected increase of IFG volumes in the middle of the bilingual experience spectrum, right after the initial decrease, which itself was followed by a decrease at the highest levels of bilingual experience. This effect might be caused by a change in the nature of the cognitive demands that bilingualism poses after the first stages of bilingual experience, and before reaching full efficiency (Pliatsikas, 2020), such as the exposure to novel bilingual naturalistic contexts, which would suppose renewed high control demands and might be accompanied by increases in IFG volume, which also seem to normalise again with increasing experience. This pattern escapes the predictions of previous models, which makes it hard to interpret in more detail. To the best of our knowledge, such an effect had not been reported before, but this might be due to the fact that previous studies with similar socio-linguistic characteristics did not use continuous non-linear approaches on cortical GM volumes. Taken at face value, this finding suggests that the dynamicity of the effects of bilingualism in immersive environments may hold even for cortical regions, not just subcortical or the cerebellum as it was previously thought (Deluca et al., 2019b; Pliatsikas, 2020) and calls for more evidence from similar samples that are highly immersed for long periods, which will help elaborate on the relevant theories.

Our results further corroborate suggestions that bilingualism increases the volume of the putamen (Abutalebi et al., 2013; Burgaleta et al., 2016; Pliatsikas et al., 2017), and that these effects may be a function of measures of bilingual experiences, such as length of immersion in the L2 (Deluca et al., 2019a), or the general degree of bilingualism (Korenar et al., 2021). This region receives inputs from parietal associative areas and is connected to motor regions (Cacciola et al., 2017), which goes in line with evidence showing its involvement in phonological processing (Tettamanti et al., 2005), language control (Hervais-Adelman et al., 2017), motor programming (Garbin et al., 2010), and articulation of L2 (Klein et al., 1994, 1995, 2006; Simmonds et al., 2011; Berken et al., 2016, 2017). Therefore, it is hypothesized that is more often recruited by bilinguals than monolinguals, leading to volume increases, since the first learn and continuously use a wider range of speech sounds than the second (Burgaleta et al., 2016), and need to control motor programmes between the two languages (Pliatsikas, 2020). Crucially, this effect might be independent of immersion, since it has been reported in immersed and non-immersed bilinguals (Deluca et al., 2019a; Korenar et al., 2021), and may be related to simultaneous acquisition and native-like accent proficiency (Berken et al., 2016).

Similar to the putamen, our results also corroborate previous evidence showing GM volume increases in the cerebellum of immersed bilinguals (Filippi et al., 2011; Pliatsikas et al., 2014; Burgaleta et al., 2016). The cerebellum is critical to language control due to its connections to the inferior frontal cortex and thalamus (Abutalebi and Green, 2016). It has also been suggested to participate in error-based learning of complex structural rules, as a part of the procedural memory system (Ullman, 2004). Notably, GM density in the cerebellum has been linked to efficiency in suppressing the first language when using in the second (Filippi et al., 2011) and cerebellar volume is directly related to the speed of processing of grammatical rules in L2 (Pliatsikas et al., 2014). All this evidence suggests that immersive bilingual environments entail high demands of language control and grammatical processing, which involves a special recruitment of the cerebellum and an increase in its volume in all stages of the immersed bilingual experience (Deluca et al., 2019b; Pliatsikas, 2020).

Some major cortical regions that lacked significant changes in our results were ACC and inferior parietal cortex. The inferior parietal lobule is thought to be crucial for the integration of semantics and phonology of recently learned vocabulary (Richardson et al., 2010), a process that might have already taken place even in our less experienced bilingual participants, since they could be considered “passive bilinguals” (Calabria et al., 2020; Costumero et al., 2020). Alternatively, the ACC is associated to conflict monitoring, which is hypothesized to be especially required in dual-language interactional contexts (Green and Abutalebi, 2013). However, in territories where Catalan and Spanish are widely used, bilinguals tend to mix both languages during the same interaction (Rodriguez-Fornells et al., 2006; Garbin et al., 2011), resulting in a bilingual experience closer to dense code-switching, where opportunistic planning is hypothesized to be more relevant for the interaction than conflict monitoring (Green and Abutalebi, 2013). Moreover, voluntary switching, as opposed to imposed by external cues, has been shown to require less ACC and prefrontal MEG activation (Blanco-Elorrieta and Pylkkänen, 2017). Since most of the population in the region where we carried out our study understands both languages, we interpret that switching is probably more natural than forced, and this could explain the absence of significant effects in the ACC as a function of bilingual experience. The fact that we found significant effects only in IFG and cerebellum cortically also goes in line with ACH predictions for dense code-switching interactional contexts, where special recruitment of these regions is expected (Abutalebi and Green, 2016). Still, we did not measure the characteristics of our participants’ conversational context, so these interpretations remain speculative. Future research should try to measure bilingualism experiences not only focusing on usage diversity, intensity, duration, and proficiency, but also on the characteristics of interactional contexts where participants make use of their languages, e.g., nature of switching practices. As for the subcortical structures described in the ACH, we did not find the expected significant changes as a function of bilingualism for the caudate nucleus and thalamus. Volumes of caudate nucleus are expected to increase in bilinguals who start acquiring vocabulary of an L2, and renormalize with increased experiences (Pliatsikas, 2020). However, previous evidence suggests that these changes are restricted to bilinguals with limited immersion, due to less proficiency and practice of L2, and would not be necessary for bilinguals in an active immersive environment, an interpretation that goes in line with the immersive context where our bilinguals find themselves and the lack of significant results we observed in this region (Pliatsikas et al., 2017). Regarding the thalamus, it is believed to intervene in the selection of relevant lexical and semantic representations in bilinguals (Abutalebi and Green, 2016), but previous studies have emphasized the specialized contribution of its nuclei to different language functions, such as naming or active speech listening, and advocated for investigating these nuclei separately (Llano, 2013; Burgaleta et al., 2016). Thus, the lack of regional subdivisions in our analyses might have masked GM volume changes in different thalamic nuclei as a function of bilingual experience.

To summarize, in this study we investigated the dynamic effects of bilingualism on GM volumes of healthy participants with a wide variety of bilingual experiences, living in a naturalistic and immersive bilingual environment. We reported a non-linear relationship between IFG and bilingualism score, a pattern that largely goes in line with predictions for effects in environments with high bilingual immersion, increased diversity and intensity of language use. We also reported linear putaminal and cerebellar GM volume increases as a function of bilingualism, which might reflect a growing need to control for motor programmes and grammatical processing. Our results further support the dynamic nature of bilingualism’s effects on brain structure and show that this dynamicity is also present in immersive environments.

## Data Availability Statement

The raw data supporting the conclusions of this article will be made available by the authors, without undue reservation.

## Ethics Statement

The studies involving human participants were reviewed and approved by Ethics Committee of the Universitat Jaume I. The patients/participants provided their written informed consent to participate in this study.

## Author Contributions

VC, CÁ, and CP contributed to conception and design of the study. VC and LM-M organized the database. LM-M performed the statistical analysis and wrote the first draft of the manuscript. All authors contributed to manuscript revision, read, and approved the submitted version.

## Conflict of Interest

The authors declare that the research was conducted in the absence of any commercial or financial relationships that could be construed as a potential conflict of interest.

## Publisher’s Note

All claims expressed in this article are solely those of the authors and do not necessarily represent those of their affiliated organizations, or those of the publisher, the editors and the reviewers. Any product that may be evaluated in this article, or claim that may be made by its manufacturer, is not guaranteed or endorsed by the publisher.

## Footnotes

1
http://dbm.neuro.uni-jena.de/cat/

2
https://github.com/cobralab/atlases

3
https://www.r-project.org/
