# Supplementary material for: Dynamic Effects of Immersive Bilingualism on Cortical and Subcortical Grey Matter Volumes
Source: Front Psychol. 2022 Apr 25;13:886222. doi: 10.3389/fpsyg.2022.886222 (PMC9109104; doi:10.3389/fpsyg.2022.886222)

Supplementary Material

1. **Bilingualism questionnaire (Spanish original)**

Código:

CUESTIONARIO DE USO DE LAS LENGUAS

Edad…………….

Lugar de nacimiento…………….

Lugar de residencia actual…………….

Si no es donde naciste, desde cuando vives en el lugar actual…………….

Lugar de nacimiento del padre…………….

Lugar de nacimiento de la madre…………….

A qué edad comenzaste a escuchar de forma continuada el catalán…………….

A qué edad comenzaste a utilizar (hablar) el catalán…………….

Cómo (dónde) aprendiste el catalán…………….

A qué edad comenzaste a escuchar de forma continuada el castellano…………….

A qué edad comenzaste a utilizar (hablar) el castellano…………….

1. Indica la lengua (catalán, castellano, ambas u otras) que usualmente utilizas para hablar con:

- Padre: Madre: Hermano/as: Novio/a:

1. Si de pequeño hablabas con tus padres o hermanos en alguna otra lengua de la que utilizas actualmente, indica a qié edad se va a producir el cambio:

- Padre: Madre: Hermano/as: Novio/a:

1. ¿Qué otras lenguas puedes utilizar (hablar, leer, escribir)? …………….

- ¿A qué edad iniciaste el aprendizaje formal de estas lenguas? …………….

Señala la opción que mejor te representa en cada una de las siguientes preguntas:

Qué nivel de comprensión tienes de estas lenguas:

Francés: perfectamente bien suficiente muy poco nada

Inglés: perfectamente bien suficiente muy poco nada

Catalán: perfectamente bien suficiente muy poco nada

Castellano: perfectamente bien suficiente muy poco nada

Qué nivel de lectura tienes de estas lenguas:

Francés: perfectamente bien suficiente muy poco nada

Inglés: perfectamente bien suficiente muy poco nada

Catalán: perfectamente bien suficiente muy poco nada

Castellano: perfectamente bien suficiente muy poco nada

Cómo hablas en estas lenguas (fluidez):

Francés: perfectamente bien suficiente muy poco nada

Inglés: perfectamente bien suficiente muy poco nada

Catalán: perfectamente bien suficiente muy poco nada

Castellano: perfectamente bien suficiente muy poco nada

Cómo hablas en estas lenguas (corrección de pronunciación):

Francés: perfectamente bien suficiente muy poco nada

Inglés: perfectamente bien suficiente muy poco nada

Catalán: perfectamente bien suficiente muy poco nada

Castellano: perfectamente bien suficiente muy poco nada

Cómo escribes en estas lenguas:

Francés: perfectamente bien suficiente muy poco nada

Inglés: perfectamente bien suficiente muy poco nada

Catalán: perfectamente bien suficiente muy poco nada

Castellano: perfectamente bien suficiente muy poco nada

¿En qué lengua te sientes más cómodo/a?

Catalán Castellano En ambas igual

¿Si desafortunadamente hubieras sufrido un accidente cerebral que te supusiera la pérdida de una lengua, cuál de las dos preferirías conservar (sin tener en cuenta criterios prácticos)?

Catalán Castellano

Si tienes (o tuvieras) un perro o un gato, en qué lengua le hablas (o hablarías)

Catalán Castellano En ambas igual

¿Percibes diferencias dialectales?

En castellano: No Sólo entre algunos dialectos Sí, claramente

En catalán: No Sólo entre algunos dialectos Sí, claramente

Evalúa el porcentaje de tiempo al día que dedicas a escuchar cada lengua (incluyendo clases, programas de TV, radio, etc)

Catalán Castellano Otras

Evalúa el porcentaje de tiempo al día que dedicas a hablar en cada lengua

Catalán Castellano Otras

En respuesta a las preguntas que vienen a continuación, señala con un círculo la frecuencia con la que utilizaste el castellano y el catalán a las diferentes edades y en las diferentes situaciones reseñadas. Para ello, utiliza la siguiente escala:

1-Solo castellano

2-Castellano frecuentemente, catalán raramente

3-Principalmente castellano, utilizando el catalán al menos una cuarta parte del tiempo

4-Uso equitativo de castellano y catalán

5-Principalmente catalán, utilizando el castellano al menos una cuarta parte del tiempo

6-Catalán frecuentemente, castellano raramente

7-Solo catalán

1. ¿Siendo un niño pequeño, antes de iniciar la etapa escolar?

- 1 2 3 4 5 6 7

2. ¿Siendo un niño, en la etapa de educación primaria?

EN LA ESCUELA

- 1 2 3 4 5 6 7

EN CASA

- 1 2 3 4 5 6 7

EN OTROS LUGARES

- 1 2 3 4 5 6 7

1. ¿En la pubertad, en la etapa de educación secundaria y en el bachillerato?

EN LA ESCUELA

- 1 2 3 4 5 6 7

EN CASA

- 1 2 3 4 5 6 7

EN OTROS LUGARES

- 1 2 3 4 5 6 7

1. ¿En la edad adulta?

EN LA UNIVERSIDAD O EN EL TRABAJO

- 1 2 3 4 5 6 7

EN CASA

- 1 2 3 4 5 6 7

EN OTROS LUGARES

- 1 2 3 4 5 6 7

1. **Bilingualism questionnaire (English translation)**

Code:

USE OF LANGUAGES QUESTIONNAIRE

Age…………….

Birthplace…………….

Current place of residence…………….

If that is not where you were born, since when do you live in that location…………….

Father’s birthplace…………….

Mother’s birthplace…………….

At what age did you start hearing Catalan continuously…………….

At what age did you start using (speaking) Catalan…………….

How (where) did you learn Catalan…………….

At what age did you start hearing Spanish continuously …………….

At what age did you start using (speaking) Spanish…………….

1. Indicate the language (Catalan, Spanish, both or others) that you normally use to speak with your:

- Father: Mother: Brother/Sister/s: Couple:

1. If you spoke a different language from the one you use now with your parents or brother/sister/s, indicate at what age did you change:

- Father: Mother: Brother/Sister/s: Couple:

1. What other languages can you use (speak, read, write)? ……….

- At what age did you start formal learning of those languages? …………….

Mark the option that represents you best in each one of the following questions:

What level of comprehension do you have of the following languages?

French: perfect good sufficient very little nothing

English: perfect good sufficient very little nothing

Catalan: perfect good sufficient very little nothing

Spanish: perfect good sufficient very little nothing

What is your reading level in the following languages?

French: perfect good sufficient very little nothing

English: perfect good sufficient very little nothing

Catalan: perfect good sufficient very little nothing

Spanish: perfect good sufficient very little nothing

How do you speak the following languages (fluency)?

French: perfect good sufficient very little nothing

English: perfect good sufficient very little nothing

Catalan: perfect good sufficient very little nothing

Spanish: perfect good sufficient very little nothing

How do you speak the following languages (pronunciation correctness)?

French: perfect good sufficient very little nothing

English: perfect good sufficient very little nothing

Catalan: perfect good sufficient very little nothing

Spanish: perfect good sufficient very little nothing

How well do you write in the following languages?

French: perfect good sufficient very little nothing

English: perfect good sufficient very little nothing

Catalan: perfect good sufficient very little nothing

Spanish: perfect good sufficient very little nothing

What language do you feel more comfortable with?

Catalan Spanish Both equally

If you unfortunately suffered from brain damage and you had to lose one language, which one would you prefer to keep (without considering practical criteria)?

Catalan Spanish

If you have (or had) a dog or a cat, what language do you (or would you) use with them?

Catalan Spanish Both equally

Do you perceive dialectal differences?

In Spanish: No Only between some dialects Yes, clearly

In Catalan: No Only between some dialects Yes, clearly

Evaluate the percentage of time in a day that you dedicate to listening to each language (including courses, TV programs, radio…)

Catalan Spanish Others

Evaluate the percentage of time in a day that you dedicate to speaking in each language

Catalan Spanish Others

In the following questions, mark with a circle the frequency of use of Spanish and Catalan at different ages and situations. To do so, use the following scale:

1-Only Spanish

2-Spanish frequently, Catalan rarely

3-Mainly Spanish, using Catalan at least ¼ of the time

4-Equal use of Spanish and Catalan

5-Mainly Catalan, using Spanish at least ¼ of the time

6-Catalan frequently, Spanish rarely

7-Only Catalan

1. When being a toddler, before starting the schooling period.

- 1 2 3 4 5 6 7

2. When being a child, at elementary school.

AT SCHOOL

- 1 2 3 4 5 6 7

AT HOME

- 1 2 3 4 5 6 7

IN OTHER ENVIRONMENTS

- 1 2 3 4 5 6 7

3. At puberty, during secondary school and high school.

AT SCHOOL

- 1 2 3 4 5 6 7

AT HOME

- 1 2 3 4 5 6 7

IN OTHER ENVIRONMENTS

- 1 2 3 4 5 6 7

4.During adulthood.

AT THE UNIVERSITY OR WORKPLACE

- 1 2 3 4 5 6 7

AT HOME

- 1 2 3 4 5 6 7

IN OTHER ENVIRONMENTS

- 1 2 3 4 5 6 7

1. **Results from the first-level models (p-values).**

**Inferior frontal gyrus**

| **Reference level of hemisphere** | **Left hemisphere** | **Right hemisphere** |
| --- | --- | --- |
| Bilingualism score | 0.064 | 0.074 |
| TIV | <0.001 | <0.001 |
| Age | <0.001 | <0.001 |
| Hemisphere | <0.001 | <0.001 |
| Bilingualism score x age | 0.479 | 0.479 |
| Bilingualism score x hemisphere | 0.417 | 0.417 |

**Parietal (supramarginal gyrus)**

| **Reference level of hemisphere** | **Left hemisphere** | **Right hemisphere** |
| --- | --- | --- |
| Bilingualism score | 0.404 | 0.578 |
| TIV | <0.001 | <0.001 |
| Age | <0.001 | <0.001 |
| Hemisphere | <0.001 | <0.001 |
| Bilingualism score x age | 0.427 | 0.427 |
| Bilingualism score x hemisphere | 0.582 | 0.582 |

**Anterior cingulate cortex**

| **Reference level of hemisphere** | **Left hemisphere** | **Right hemisphere** |
| --- | --- | --- |
| Bilingualism score | 0.864 | 0.8807 |
| TIV | <0.001 | <0.001 |
| Age | 0.022 | <0.028 |
| Hemisphere | <0.001 | <0.001 |
| Bilingualism score x age | 0.819 | 0.819 |
| Bilingualism score x hemisphere | 0.553 | 0.553 |

**Motor cortex**

| **Reference level of hemisphere** | **Left hemisphere** | **Right hemisphere** |
| --- | --- | --- |
| Bilingualism score | 0.568 | 0.857 |
| TIV | <0.001 | <0.001 |
| Age | <0.001 | <0.001 |
| Hemisphere | 0.136 | 0.136 |
| Bilingualism score x age | 0.536 | 0.341 |
| Bilingualism score x hemisphere | 0.451 | 0.387 |

**Premotor cortex**

| **Reference level of hemisphere** | **Left hemisphere** | **Right hemisphere** |
| --- | --- | --- |
| Bilingualism score | 0.243 | 0.199 |
| TIV | <0.001 | <0.001 |
| Age | <0.001 | <0.001 |
| Hemisphere | <0.001 | <0.001 |
| Bilingualism score x age | 0.067 | 0.067 |
| Bilingualism score x hemisphere | 0.379 | 0.379 |

**Thalamus**

| **Reference level of hemisphere** | **Left hemisphere** | **Right hemisphere** |
| --- | --- | --- |
| Bilingualism score | 0.945 | 0.394 |
| TIV | <0.001 | <0.001 |
| Age | <0.001 | <0.001 |
| Hemisphere | <0.001 | <0.001 |
| Bilingualism score x age | 0.885 | 0.885 |
| Bilingualism score x hemisphere | 0.352 | 0.352 |

**Caudate**

| **Reference level of hemisphere** | **Left hemisphere** | **Right hemisphere** |
| --- | --- | --- |
| Bilingualism score | 0.913 | 0.838 |
| TIV | <0.001 | <0.001 |
| Age | <0.001 | <0.001 |
| Hemisphere | <0.001 | <0.001 |
| Bilingualism score x age | 0.552 | 0.552 |
| Bilingualism score x hemisphere | 0.848 | 0.848 |

**Putamen**

| **Reference level of hemisphere** | **Left hemisphere** | **Right hemisphere** |
| --- | --- | --- |
| Bilingualism score | 0.849 | 0.7961 |
| TIV | <0.001 | <0.001 |
| Age | <0.001 | <0.001 |
| Hemisphere | 0.64 | 0.64 |
| Bilingualism score x age | 0.308 | 0.308 |
| Bilingualism score x hemisphere | 0.890 | 0.890 |

**Cerebellum**

| **Reference level of hemisphere** | **Left hemisphere** | **Right hemisphere** |
| --- | --- | --- |
| Bilingualism score | 0.488 | 0.493 |
| TIV | <0.001 | <0.001 |
| Age | <0.001 | <0.001 |
| Hemisphere | <0.001 | <0.001 |
| Bilingualism score x age | 0.947 | 0.947 |
| Bilingualism score x hemisphere | 0.965 | 0.965 |

**Insula**

| **Reference level of hemisphere** | **Left hemisphere** | **Right hemisphere** |
| --- | --- | --- |
| Bilingualism score | 0.877 | 0.743 |
| TIV | <0.001 | <0.001 |
| Age | <0.001 | <0.001 |
| Hemisphere | 0.209 | 0.209 |
| Bilingualism score x age | 0.988 | 0.988 |
| Bilingualism score x hemisphere | 0.736 | 0.736 |

1. **Assessment of model fits**

| **ROI** | **Smooth term** | **K (number of basis functions)** | **Estimated degrees of freedom** | **k-index** | **p-value of significant patterns in residuals** |
| --- | --- | --- | --- | --- | --- |
| **IFG** | **Bilingualism score** | 8 | 3.84 | 0.91 | 0.22 |
| **Putamen** | **Age** | 2 | 1.52 | 0.99 | 0.47 |

# Range and distribution of bilingual scores


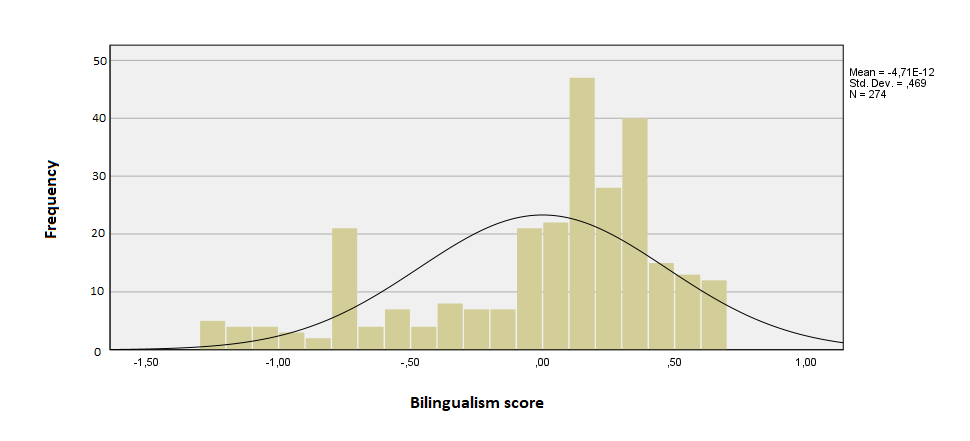

Supplement: Supplementary file 1 [file Data_Sheet_1.docx]
